# Engineering Nutritional Profiles in Edible Insects Through Diet–Microbiome Interactions: Mechanisms, Applications and Future Directions

**DOI:** 10.3390/insects17080842

**Published:** 2026-08-14

**Authors:** Mohamed Ezzaitouni, Tarik Chileh Chelh, El-Hassan Belarbi, José Luis Guil-Guerrero

**Affiliations:** 1 Tecnología de Alimentos, Universidad de Almería, 04120 Almería, Spain; chileh@hotmail.es (T.C.C.); jlguil@ual.es (J.L.G.-G.); 2 Department of Chemical Engineering, University of Almería, 04120 Almería, Spain; ebelarbi@ual.es

**Keywords:** bioconversion, microbiome, lipidomics, metabolomics, bioengineering

## Abstract

The growing demand for sustainable and nutritionally optimized protein sources has positioned edible insects as promising platforms for next-generation food and feed systems. Although insect composition varies with diet, the deliberate engineering of their nutritional profiles through controlled dietary and microbiome modulation remains insufficiently characterized. This review proposes an integrative framework in which edible insects are conceptualized as programmable biological systems whose biochemical composition can be tailored through diet–microbiome interactions. Current evidence is critically synthesized to elucidate how dietary inputs, including agro-industrial by-products and algal biomass, reshape gut microbial communities and metabolic pathways in key species such as *Hermetia illucens*, *Tenebrio molitor*, and *Acheta domesticus*. Emphasis is placed on engineering lipid metabolism and fatty acid composition because of their relevance to human nutrition, animal nutrition, and the development of functional food and feed products. By integrating insights from insect physiology, microbiology, and nutritional biochemistry, this work outlines strategies for precision feeding and microbiome-guided interventions aimed at optimizing insect-derived biomass. Key knowledge gaps and technical limitations are also identified, highlighting the need to transition from empirical approaches toward predictive, systems-based nutritional engineering. Collectively, this review highlights the transition from empirical feeding strategies toward predictive, systems-based approaches for designing customized insect biomass within sustainable circular bioeconomy frameworks.

## Simple Summary

The growing demand for sustainable sources of food and animal feed has increased interest in edible insects, which can convert low-value organic materials into nutritious biomass. However, their nutritional composition can vary depending on what they eat and how they are reared, making it difficult to consistently produce insects with desired nutritional properties. This review examines how diet and the communities of microorganisms living in the insect gut work together to influence growth, nutrient use, fats, proteins, and other valuable compounds. The available evidence shows that diets containing agricultural by-products, food residues, seaweeds, and other nutrient-rich materials can change the nutritional composition and quality of edible insects. The review also shows that gut microorganisms contribute to digestion, nutrient transformation, adaptation to different diets, and the conversion of organic materials into useful biomass. However, important gaps remain because the mechanisms behind these interactions are not yet fully understood and production methods are not sufficiently standardized. Combining improved feeding strategies, knowledge of gut microorganisms, biological analysis, and automated production could help produce insects with more predictable nutritional properties. This approach could support sustainable food and feed production, reduce organic waste, and strengthen circular and resource-efficient agricultural systems.

## 1. Introduction

The increasing global demand for protein-rich foods is placing considerable pressure on conventional livestock systems, which are associated with high greenhouse gas emissions, extensive land use, and significant freshwater consumption [1]. Consequently, sustainable dietary strategies capable of balancing nutritional requirements with environmental sustainability have become a global priority [2]. Although insects have been consumed traditionally in many regions of the world for centuries, their scientific recognition as a sustainable food resource was first highlighted by [3], who proposed edible insects as a potential solution to global food shortages. Since then, edible insects have attracted increasing scientific and industrial attention as additional sources of proteins, lipids, vitamins, minerals, and bioactive compounds that can contribute to more sustainable food and feed systems while reducing pressure on conventional livestock production [2,3]. Among the approximately 2000 edible insect species reported worldwide, *Hermetia illucens*, *Tenebrio molitor*, and *Acheta domesticus* have emerged as the principal species for industrial production because of their high feed conversion efficiency, rapid growth, and capacity to convert low-value organic substrates into nutrient-rich biomass [4,5,6,7,8,9]. Beyond their nutritional value, these insects represent efficient biological platforms for the valorization of agricultural by-products and organic waste streams, supporting resource recovery and circular bioeconomy strategies [5,8]. Their favorable protein and lipid composition, combined with relatively low environmental impacts compared with conventional livestock, has stimulated increasing interest in their application in both food and animal feed systems [6,7,9].

A distinctive characteristic of edible insects is their remarkable nutritional plasticity. Their biochemical composition is influenced by multiple interacting factors, including species, developmental stage, diet composition, processing methods, and rearing conditions. Consequently, dietary interventions have become one of the most promising approaches for tailoring insect biomass. Feeding strategies based on omega-3-rich ingredients, agro-industrial by-products, seaweeds, olive pomace, and former foodstuffs have demonstrated their capacity to modify protein content, lipid accumulation, fatty acid (FA) profiles, growth performance, antioxidant status, and the concentration of bioactive compounds [6,10,11]. Previous reviews have emphasized that insect nutritional quality results from the interaction of biological and environmental factors rather than diet alone [12]. Likewise, nutrient composition, chitin content, and overall nutritional quality vary according to developmental stage and species, with several edible insects exhibiting nutrient profiles comparable to conventional animal-derived foods [13,14]. In parallel with dietary manipulation, the insect gut microbiome has emerged as a key regulator of nutrient digestion, metabolic homeostasis, and adaptation to diverse feeding substrates. In *H. illucens*, gut microbial communities are strongly influenced by diet composition, developmental stage, gut compartment, and rearing conditions, directly affecting nutrient transformation, substrate utilization efficiency, and biomass quality [15,16,17,18,19,20,21,22]. These findings highlight the importance of microbiome-mediated processes in precision nutritional engineering. These interacting determinants have been comprehensively reviewed by Meyer-Rochow et al. [23], who highlighted that species, developmental stage, sex, diet, environmental conditions, and processing methods collectively shape the nutritional quality and chemical composition of edible insects.

Despite the rapid expansion of edible insect research, important knowledge gaps remain. Most studies investigated nutritional composition, dietary substrates, gut microbiota, or bioconversion efficiency independently, whereas integrated analyses linking diet, microbiome dynamics, metabolic regulation, and biomass quality remain scarce [24]. Furthermore, methodological heterogeneity limits direct comparisons among studies and hinders the establishment of predictive nutritional strategies. Additional challenges include industrial scalability, substrate safety, regulatory frameworks, consumer acceptance and the limited understanding of nutrient bioavailability, long-term safety, and anti-nutritional compounds [4,25]. Addressing these limitations will be essential for advancing edible insects from promising biological resources to predictable and customizable production platforms.

**Figure 1:**
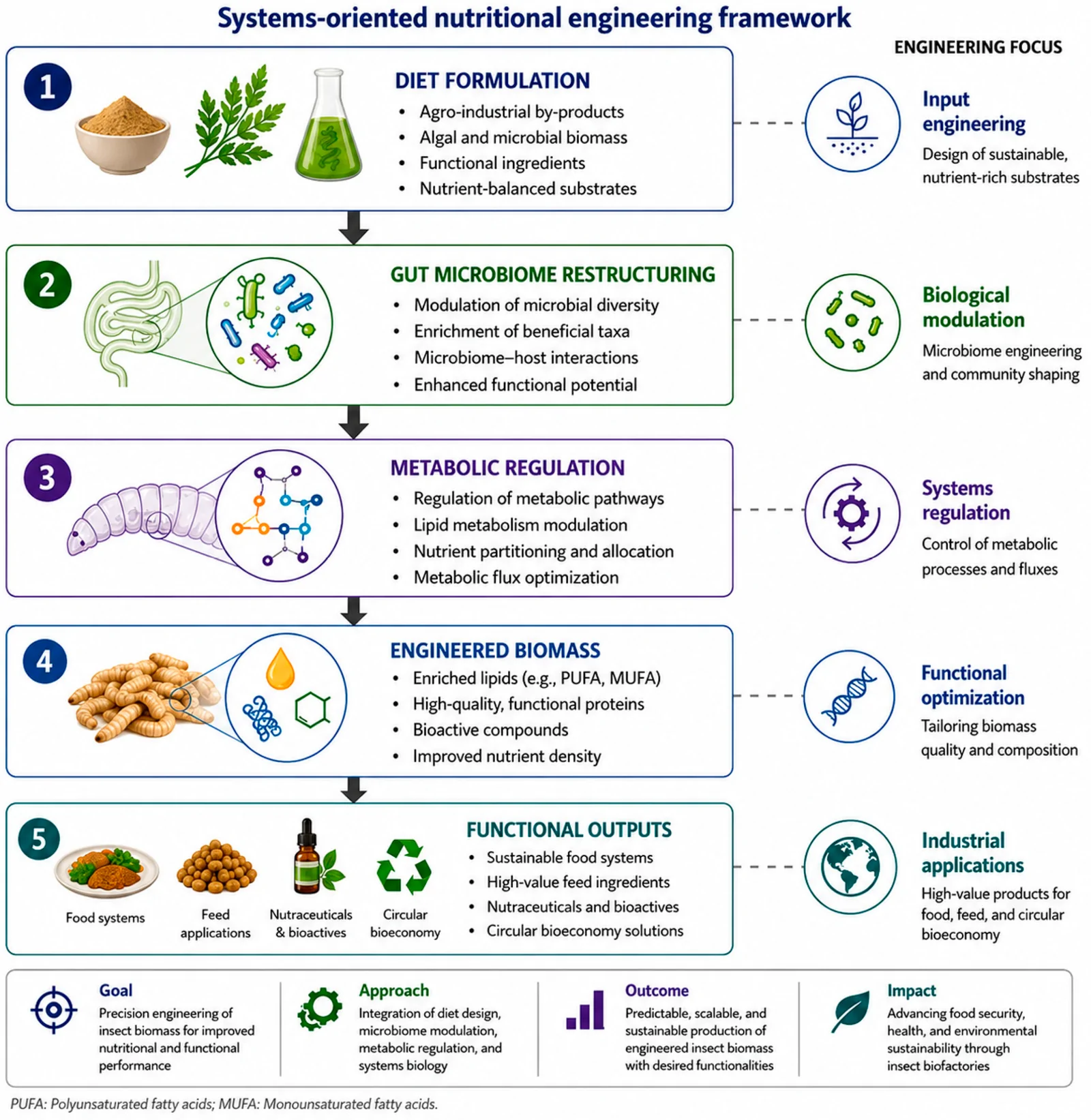
Conceptual framework of diet–microbiome-driven biomass engineering in edible insects. The conceptual framework is based on the experimental evidence discussed in Section 2, Section 3, Section 4, Section 5 and Section 6 of the manuscript.

Against this background, the present review provides a comprehensive and critical synthesis of current knowledge on diet–microbiome interactions and their role in shaping the nutritional composition of edible insects. Particular emphasis is placed on understanding how dietary interventions regulate gut microbial communities, metabolic pathways, lipid metabolism, and biomass quality in economically relevant species. Beyond summarizing the current evidence, this review identifies major technological, regulatory, and scientific challenges and proposes a systems-based framework for precision feeding and microbiome-guided biomass engineering. A conceptual framework illustrating the integration of diet formulation, gut microbiome restructuring, metabolic regulation, and engineered insect biomass production is presented in Figure 1.

## 2. Nutritional Plasticity and Biomass Engineering in Edible Insects

### 2.1. Nutritional Composition and Metabolic Flexibility Across Edible Insect Species

The nutritional composition of edible insects varies considerably among species and is influenced by multiple interacting factors, including developmental stage, diet composition, environmental conditions, rearing practices, and processing methods [26]. Although this variability complicates direct comparisons among studies, it also highlights the remarkable metabolic flexibility of edible insects and their potential as customizable sources of nutrients for sustainable food and feed systems. Comparative studies show that several edible insect species contain high-quality proteins, favorable FA profiles, and significant amounts of vitamins, minerals, and bioactive compounds [27].

Beyond their nutritional value, edible insects provide bioactive peptides, chitin, and antioxidant compounds, while processing technologies such as drying, fermentation, extrusion, and protein extraction influence nutrient stability, digestibility, techno-functional properties, sensory quality, and shelf life [28]. Likewise, protein-rich powders produced from edible crickets have demonstrated high nutritional density together with diverse peptide profiles, reinforcing their potential as ingredients for functional foods and alternative protein formulations [29]. Nevertheless, processing conditions may also contribute to variations in protein functionality and nutrient bioavailability, emphasizing the importance of standardized processing protocols for industrial applications.

Edible insects exhibit substantial variability in macronutrient composition, particularly regarding proteins, lipids, and carbohydrates, reflecting their remarkable physiological adaptability [30]. Protein represents one of the major nutritional components of edible insects, with several species presenting balanced amino acid profiles comparable to those of conventional livestock. Lipid composition, however, is considerably more dynamic and responds rapidly to dietary manipulation, providing opportunities to tailor FA profiles and improve nutritional quality through targeted feeding strategies [31]. In addition to their nutritional role, insect-derived proteins possess valuable techno-functional properties, including emulsifying capacity, foaming ability, water-holding capacity, and gel formation, which support their incorporation into diverse food formulations [32]. Despite these advantages, variability in species, developmental stage, substrates, rearing conditions, and processing methods limits direct comparisons among studies, emphasizing the need for standardized analytical methodologies and harmonized reporting criteria. Table 1 illustrates the considerable variability in nutritional composition of edible insects, highlighting species-specific differences in proteins, lipids, and micronutrients.

### 2.2. Dietary Modulation of Nutritional Profiles

Diet composition is one of the principal determinants of the nutritional quality of edible insects, directly influencing growth performance, nutrient utilization, and the accumulation of proteins, lipids (FAs), and bioactive compounds [40]. Dietary manipulation using agro-industrial by-products, vegetable residues, former foodstuffs, and marine biomass consistently improves insect nutritional quality while supporting waste valorization. The magnitude of these responses varies among insect species and substrates due to differences in digestive physiology, nutrient availability, and rearing conditions. For example, *H. illucens* larvae reared on fish offal and seaweed mixtures accumulated higher levels of nutritionally valuable polyunsaturated fatty acids (PUFAs) [41]. Overall, current evidence supports dietary engineering as an effective strategy for tailoring insect biomass, although direct comparisons among studies remain constrained by differences in experimental design and analytical methodologies.

### 2.3. Engineering Lipid Metabolism and FA Profiles

As summarized in Table 2, dietary interventions consistently modify fatty acid composition across edible insect species. Although marine-derived substrates generally increase PUFA accumulation, plant-based substrates mainly influence total lipid content and specific fatty acid classes, illustrating the diversity of nutritional responses observed under different feeding strategies. Lipid metabolism has attracted increasing attention due to its relevance for nutritional quality and functional food applications. Black soldier fly larvae (*H. illucens*) exhibit remarkable metabolic flexibility, allowing the modulation of FA composition through dietary interventions and substrate formulation [35]. However, diet is only one of several factors regulating lipid metabolism, which is also influenced by species, developmental stage, sex, environmental conditions, and processing methods [23].

Beyond compositional changes, engineering insect lipid metabolism has important implications for food and feed applications. Improved FA profiles can enhance the nutritional quality of insect-derived ingredients and influence the fatty acid composition of animal products when enriched insects are incorporated into aquaculture diets [42].

Precision nutritional engineering therefore provides an effective framework for tailoring insect lipid composition through targeted substrate formulation [43]. Although dietary manipulation successfully modifies nutrient composition [34,44], current approaches remain largely empirical because the molecular mechanisms regulating lipid metabolism are still poorly understood. Recent studies using alternative substrates such as *Rugulopteryx okamurae* [45], with advances in transcriptomics, metabolomics, lipidomics, and functional genomics [46,47,48,49], are beginning to reveal these mechanisms and may support the development of predictive nutritional engineering strategies.

**Table 2:** Dietary interventions and FA modulation in edible insects.

Species	Lipid (%)	Major FAs	Functional/Bioactive Components	Main Applications	References
*T. molitor*	Flaxseed-enriched substrates	Increased α-linolenic acid and total PUFA	Improved omega-3 profile	Nutritional tailoring of functional biomass	[14]
*T. molitor*	Algal biomass supplementation	Enhanced long-chain PUFA accumulation	Increased nutritional value	Sustainable lipid engineering	[18]
*A. domesticus*	Functional plant-based diets	Modulation of MUFA/SFA balance	Improved lipid quality	Precision feeding strategies	[50]
*H. illucens*	Fish offal and seaweed mixtures	Increased omega-3 FA and altered lipid composition	Enhanced functional lipid profile	Waste-to-biomass bioconversion	[41]
*H. illucens*	Seaweed-enriched waste streams	Increased PUFA incorporation	Functional biomass enrichment	Circular bioeconomy applications	[51]
*H. illucens*	Agro-industrial by-products	Modulated lauric acid and saturated FA levels	Optimized lipid metabolism	Substrate engineering	[40]
*A. diaperinus*	Nutrient-enriched cereal substrates	Altered FA composition and lipid deposition	Improved nutritional functionality	Targeted metabolic modulation	[37]
*Gryllus bimaculatus*	Functional supplementation diets	Enhanced unsaturated FA accumulation	Bioactive lipid production	Engineered nutraceutical biomass	[31]

### 2.4. Precision Nutritional Engineering Strategies

Precision nutritional engineering integrates dietary formulation with insect physiology to produce biomass tailored for specific food and feed applications [43].

Beyond improving nutrient composition, these strategies contribute to the development of sustainable production systems by enhancing nutrient conversion efficiency and expanding the use of edible insects as alternative protein sources for animal nutrition [52,53]. Overall, precision nutritional engineering provides an effective framework for developing sustainable food and feed systems.

## 3. Gut Microbiome and Metabolic Regulation

### 3.1. Diversity and Structure of Insect Gut Microbiota

The gut microbiota plays a central role in digestion, nutrient transformation, metabolic regulation and adaptation to different dietary substrates in edible insects. In *H. illucens*, intestinal microbial communities exhibit considerable diversity and are strongly influenced by diet composition, suggesting close interactions between feeding substrates and microbial structure [16]. Moreover, microbial composition varies among different gut regions, indicating the presence of specialized microbial functions within the digestive tract.

Current investigations have demonstrated that the microbiota of *H. illucens* is dynamically structured according to gut compartment, developmental stage and diet [15]. These microbial communities contribute to the degradation of complex organic materials and nutrient bioconversion processes, thereby enhancing substrate utilization efficiency and insect growth performance [22]. These findings emphasize the contribution of gut microbiota to nutrient utilization and metabolic performance in edible insects.

Variations in microbiota composition have been observed in *H. illucens* larvae reared on different organic waste streams and at different production scales, emphasizing the impact of substrate characteristics and environmental factors on microbial ecology [21]. Furthermore, investigations into edible insects commercialized in the European Union have revealed diverse microbial communities associated with different insect species and processing conditions [54]. However, the functional roles of many microbial taxa and their direct contribution to host metabolism remain only partially understood.

### 3.2. Functional Roles of Gut Microbiota

The gut microbiota plays essential roles in insect physiology by supporting nutrient digestion, metabolic regulation, immune function, and adaptation to different diets and environments [55]. In *H. illucens*, microbial community dynamics influence larval development and substrate conversion efficiency, while metagenomic analyses have identified genes involved in carbohydrate metabolism, amino acid biosynthesis, lipid transformation, and the degradation of complex substrates [17,56].

Beyond its physiological functions, the gut microbiome represents a promising target for microbiome-based strategies aimed at improving insect health, substrate utilization, and sustainable production systems [57]. The identification of a relatively stable core microbiome in *H. illucens* suggests that conserved microbial taxa may play key roles in insect metabolism, although their specific functions still require experimental validation [58].

### 3.3. Diet-Induced Microbiome Restructuring

Diet composition is one of the main factors driving gut microbiome restructuring in edible insects. In *H. illucens* larvae, intestinal microbial communities are strongly influenced by feeding substrates and vary across different gut regions, indicating dynamic interactions between diet composition and microbial ecology [16].

Vegetable and fruit by-products have been successfully evaluated as rearing substrates for *H. illucens* larvae, demonstrating the adaptability of the insect microbiome to diverse nutritional environments [59]. Similarly, substrate composition has been shown to influence growth efficiency, waste reduction capacity, and the chemical composition of *H. illucens* larvae [40].

Recent studies have demonstrated that nutrient formulation strategies based on protein and carbohydrate content can improve biowaste conversion efficiency and larval performance during *H. illucens* cultivation [60]. Notably, post-harvest treatments such as starvation and rinsing can modify the microbiome composition and reduce spoilage-associated bacteria in *H. illucens* larvae, highlighting the dynamic nature of insect-associated microbial communities even after rearing [37].

### 3.4. Microbiome-Mediated Metabolic Fluxes

**Figure 2:**
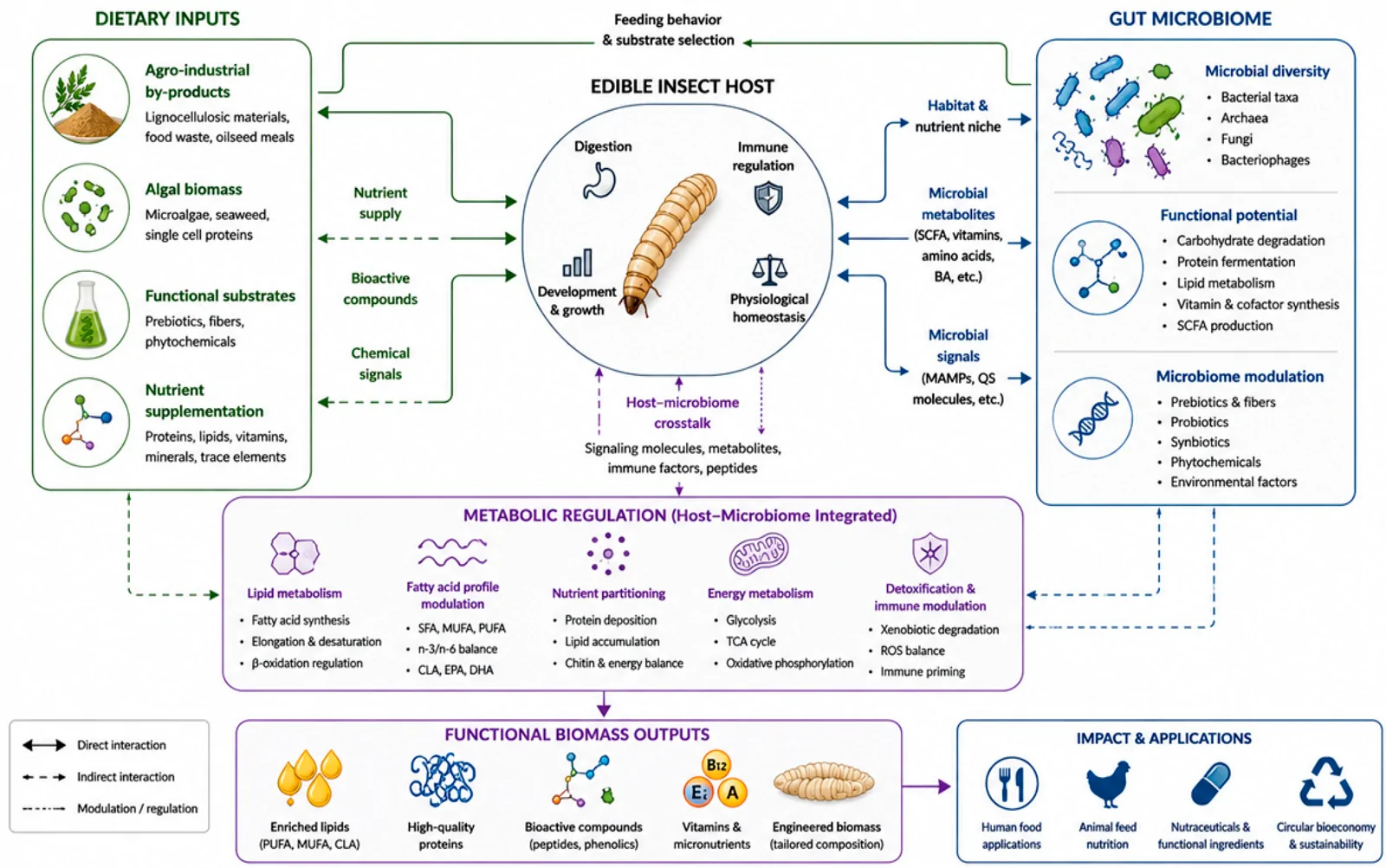
Host–diet–microbiome interaction networks regulating functional biomass engineering in edible insects. The conceptual framework is based on the experimental evidence discussed in the corresponding section of the manuscript [15,16,17,55,56,57,58].

Figure 2 illustrates how dietary composition influences gut microbial communities, which in turn regulate nutrient metabolism, growth performance, and biomass quality. The figure also highlights the reciprocal interactions between host physiology and microbial functions that collectively determine the efficiency of nutritional engineering strategies. The gut microbiome of edible insects plays a central role in metabolic regulation by contributing to nutrient transformation, biodegradation processes, detoxification mechanisms, and substrate utilization efficiency. In *H. illucens* larvae, intestinal microbial communities are closely associated with metabolic pathways involved in the degradation of complex organic compounds and nutrient recycling [22]. These microbial interactions contribute significantly to larval growth performance and bioconversion capacity.

Newly available omics data have demonstrated that gut-associated microorganisms actively enhance food waste biodegradation by strengthening metabolic functions related to organic matter decomposition and nutrient assimilation [61]. Similarly, co-conversion systems integrating *H. illucens* larvae and functional bacteria have shown improved efficiency in transforming biowaste into protein-rich feed and organic fertilizers, highlighting the synergistic relationship between insect hosts and associated microbiota [62]. The microbiome also plays an important role in detoxification and environmental adaptation. For example, intestinal microbial communities have been associated with the degradation of aflatoxin B1 in *H. illucens* larvae, suggesting that microbiome-mediated detoxification pathways contribute to insect resilience under contaminated feeding conditions [63]. Importantly, recent investigations into transgenerational microbiome dynamics have shown that gut microbial composition can be influenced by novel substrates across successive generations, indicating long-term microbial adaptation and inheritance patterns in *H. illucens* [24].

### 3.5. Multi-Omics and Systems Biology Approaches

Genomic analyses of *H. illucens* have identified genes associated with nutrient conversion, metabolic regulation, and waste biodegradation, supporting its potential as a sustainable bioconversion organism [64]. Similarly, transcriptomic and metabolomic studies revealed that different food waste substrates can induce adaptive metabolic and physiological responses in *H. illucens* larvae [65].

Omics-based approaches have also been applied to evaluate the nutritional and functional properties of insect-derived products. *T. molitor* flour subjected to in vitro digestion showed potential interactions with the human gut microbiota, suggesting possible microbiome-modulating effects of edible insects [66]. Furthermore, processing methods can significantly affect the nutritional quality and bioavailability of nutrients in *H. illucens* larvae [67]. The increasing development of edible insect farming further highlights the potential of insects as sustainable bioconversion systems and emerging sources of food and feed [68].

Nevertheless, despite these advances, integrated multi-omics analyses linking microbiome composition with host metabolic pathways remain limited, restricting the development of predictive nutritional engineering strategies. The main microbial taxa, metabolic functions, and diet-related microbiome responses reported across selected edible insect species are summarized in Table 3.

## 4. Diet–Microbiome Interactions as Biomass Engineering Tools

### 4.1. Host–Diet–Microbiome Axis

The host–diet–microbiome axis influences nutrient utilization in edible insects, however, the metabolic mechanisms underlying these interactions remain poorly resolved. In *H. illucens*, gut microbial communities are strongly influenced by diet composition, gut region, and developmental stage, demonstrating the dynamic nature of insect-associated microbiota [15,16]. Despite these advances, most studies remain descriptive, while the mechanistic relationships linking dietary substrates, microbial dynamics, and host metabolism are still poorly understood.

Recent investigations suggest that *H. illucens* larvae can actively modify both gut and substrate microbiota depending on substrate composition and larval density [71]. These microbial communities contribute to the degradation of complex organic substrates through specialized enzymatic activities [72]. While metagenomic analyses have identified functional traits associated with nutrient conversion and larval performance [56]; however, establishing direct links between microbiome function, host metabolism, and nutritional outcomes remains a major challenge for developing predictive nutritional engineering strategies.

### 4.2. Metabolic Engineering Through Precision Feeding

Dietary enrichment with omega-3-rich substrates increases the accumulation of beneficial PUFAs in edible insects, whereas nutrient composition also influences growth performance and biomass quality [14,62].

**Figure 3:**
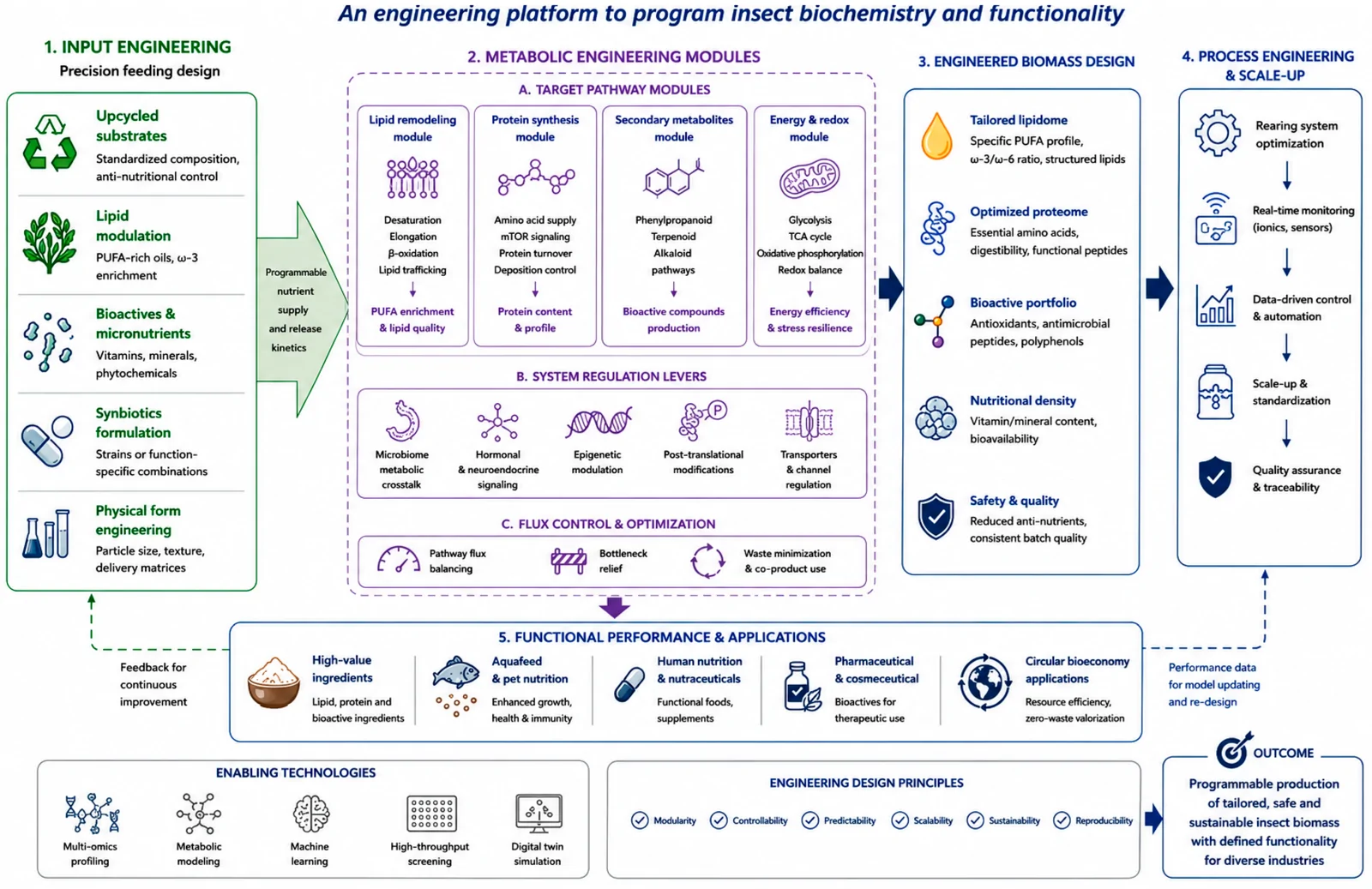
Metabolic engineering framework linking precision feeding to tailored functional biomass production in edible insects. The conceptual framework is based on the experimental evidence discussed in the corresponding section of the manuscript [14,35,43,44,45,46,47,48,49,62,64,65].

Alternative substrates, including olive pomace and former foodstuffs, further modify growth, antioxidant status, and biochemical composition, demonstrating the versatility of precision feeding strategies [10,11,40]. Although these approaches effectively modify biomass composition, the metabolic mechanisms linking dietary inputs, microbiome dynamics, and nutrient assimilation remain incompletely understood. Figure 3 illustrates the main pathways through which dietary substrates influence fatty acid biosynthesis and lipid accumulation. It also highlights the contribution of gut microbiota and molecular regulation to nutritional engineering strategies.

### 4.3. Microbiome-Guided Biomass Optimization

Studies on *H. illucens* larvae have identified relatively stable core gut microbial communities associated with nutrient metabolism and substrate utilization, suggesting that specific microbial taxa may contribute to optimized larval performance [58]. Moreover, microbial community dynamics during insect rearing can directly influence production efficiency and biomass quality [17]. Recent evidence indicates that microbial interactions and metabolic pathways play important roles in regulating nutrient assimilation and bioconversion efficiency in edible insects [71]. Furthermore, microbial-driven biowaste valorization systems integrating insects and beneficial microorganisms have demonstrated considerable potential for scalable and sustainable biomass production [73].

Microbiome modulation may also influence animal performance and gut health when insect-derived products are incorporated into feed systems. For example, housefly larvae meal affected growth performance, metabolic energy, gut health, and microbiota composition in broilers [74]. However, functional evidence linking microbial communities with biomass optimization remains limited, highlighting the need for integrated analyses of microbiome activity, host metabolism, and nutritional performance.

### 4.4. Engineering Functional Lipids and Bioactive Compounds

In *H. illucens*, lipid metabolism is strongly influenced by dietary substrates, allowing the modulation of FA profiles and the accumulation of functional lipids through nutritional strategies [36]. Comparative studies have also shown substantial variability in FA composition among edible insect species, highlighting their metabolic flexibility and nutritional potential [39].

In addition to functional lipids, edible insects contain antimicrobial peptides, phenolic compounds, and other bioactive metabolites with potential health-promoting properties [75]. Novel antimicrobial peptide genes identified in *H. illucens* demonstrate promising applications in biotechnology and antimicrobial development [76]. Likewise, *H. illucens* has been recognized as a sustainable source of proteins, lipids, and bioactive compounds with considerable industrial relevance [35]. Despite growing interest in engineering insect-derived bioactive compounds, the metabolic pathways regulating lipid biosynthesis, bioactive compound accumulation, and microbiome-mediated processes remain incompletely understood, limiting the development of predictive engineering strategies.

### 4.5. Systems-Based Optimization and Predictive Modeling

Systems-based optimization integrates genomic, metabolic, and microbiome information to improve substrate utilization, production efficiency and biomass quality in edible insects [64,65,77]. These approaches provide the basis for predictive models capable of supporting industrial-scale production and sustainable bioconversion [68,78]. Future research should integrate omics technologies, microbiome analyses, and computational modeling to develop predictive frameworks capable of optimizing insect production, nutritional quality, and industrial scalability.

## 5. Precision Feeding and Bioprocess Engineering

### 5.1. Precision Feeding Strategies

Precision feeding strategies are increasingly used to optimize growth performance, nutrient composition, and functional quality in edible insects through targeted substrate formulation. Dietary manipulation effectively modifies lipid metabolism and fatty acid (FA) composition, particularly when omega-3-rich ingredients are incorporated into insect diets [14]. Similarly, nutrient levels in feeding substrates significantly influence larval growth and biochemical composition in *H. illucens* larvae [62].

Likewise, substrate formulation plays a key role in determining growth performance, nutrient accumulation, and waste conversion efficiency in *Hermetia illucens* [40]. Supplementation with fish offal and seaweed mixtures further improves FA profiles by increasing beneficial PUFAs [41], while olive pomace supplementation enhances growth performance and nutritional quality in *Tenebrio molitor* [11]. Despite these advances, most studies remain focused on compositional outcomes rather than the metabolic and microbiome-related mechanisms underlying dietary responses. Furthermore, differences in insect species, substrate composition, and rearing conditions limit direct comparisons among studies. Future research should therefore prioritize standardized experimental protocols and mechanistic approaches to improve the predictability and scalability of precision feeding strategies for edible insect production.

### 5.2. Agro-Industrial Waste Valorization

*H. illucens* larvae efficiently convert agro-industrial residues into high-value biomass, supporting circular bioeconomy strategies and the production of sustainable feed ingredients, lipids, and biofuels [5,79].

A wide range of agro-industrial substrates, including vegetable and fruit by-products, support efficient larval development and produce nutritionally valuable biomass [34,59]. Optimizing substrate composition, particularly protein and carbohydrate levels, further improves waste conversion efficiency and insect productivity [60].

Nevertheless, variability in substrate composition, microbial safety, and the lack of standardized rearing protocols continue to limit industrial implementation, emphasizing the need for more robust production systems.

### 5.3. Algal Biomass Applications

Algal biomass represents a promising sustainable substrate for edible insect production because it provides renewable nutrients while supporting circular bioeconomy strategies. Beyond improving insect nutrition, algae can be incorporated into waste valorization systems that enhance resource efficiency and reduce dependence on conventional feed ingredients [18]. Recent studies using food waste and algal-based substrates have demonstrated improvements in larval growth, nutrient conversion, and bioconversion efficiency [80]. However, the industrial implementation of algal biomass remains constrained by economic feasibility, substrate availability, and limited knowledge of microbiome-mediated responses under large-scale production conditions.

### 5.4. Bioprocess Engineering and Industrial Scalability

Bioprocess engineering plays a key role in improving the sustainability and industrial scalability of edible insect production. Recent technological advances have focused on optimizing rearing systems, environmental control, and processing efficiency to increase biomass yield, substrate conversion, and production stability under industrial conditions [8,35,81]. However, large-scale implementation remains constrained by process standardization, automation, microbial safety, and economic feasibility, while regulatory and operational variability continue to limit broader industrial adoption [7,68].

### 5.5. Circular Bioeconomy Integration

Edible insects contribute to circular bioeconomy systems by converting low-value organic substrates into nutrient-rich biomass with high resource-use efficiency [4]. Their integration into sustainable production systems is further strengthened by advances in processing technologies, omics tools, and the recovery of high-value bioactive compounds [25,28,82].

Yet, large-scale implementation remains constrained by regulatory frameworks, substrate safety, economic feasibility, and production standardization, limiting the development of robust and predictive circular production systems [9]. In addition, inconsistencies in substrate composition and metabolic responses continue to limit predictive scalability across industrial production systems. Future research should prioritize standardized methodologies, integrated multi-omics analyses, regulatory harmonization, and industrial validation to enable predictive biomass engineering.

## 6. Industrial and Functional Applications of Engineered Insect Biomass

### 6.1. Food Systems and Functional Foods

Edible insects are increasingly recognized as sustainable ingredients for food systems because they provide high-quality proteins, lipids, vitamins, minerals, and bioactive compounds with a lower environmental impact than conventional animal proteins [27,30].

Beyond their nutritional composition, edible insects exhibit important techno-functional properties relevant to food formulation. Variability in techno-functional properties among insect proteins still limits formulation reproducibility and processing stability across industrial food systems. Insect-derived proteins exhibit characteristics such as emulsifying capacity, gel formation, and water-holding ability, which may support their incorporation into processed foods and functional formulations [32]. Processing technologies, including drying, extraction and fermentation, also influence nutritional quality, digestibility, sensory attributes, and the shelf stability of insect-based products [28]. Furthermore, sensory acceptance and consumer perception remain important factors affecting the integration of edible insects into mainstream food systems [33].

Despite growing interest in insect-based functional foods, challenges related to standardization, allergenicity, consumer acceptance, and regulatory approval continue to limit their commercial adoption. In addition, interactions between insect-derived proteins, lipid fractions, and food matrices remain insufficiently characterized under industrial processing conditions, restricting the development of standardized functional food formulations.

### 6.2. Animal Feed Applications

Edible insects are increasingly incorporated into animal feed systems due to their efficient nutrient conversion, adaptable biochemical composition, and lower environmental impact compared with conventional protein sources [34]. Among them, *Hermetia illucens* is particularly relevant due to its rapid biomass production and balanced protein and lipid composition [9]. Insect-derived ingredients have demonstrated the capacity to partially replace fishmeal and soybean meal in poultry, livestock, and aquaculture diets while maintaining feed efficiency and animal performance [82], and their bioactive compounds may contribute to gut health, metabolic regulation, and overall nutritional quality [52,53].

Nevertheless, substrate-dependent compositional variability and species-specific metabolic responses continue to limit feed standardization, while evidence linking nutritional composition with microbiome-mediated mechanisms and long-term industrial performance remains limited [9].

### 6.3. Nutraceutical and Pharmaceutical Potential

Edible insects have emerged as relevant sources of nutraceutical and pharmaceutical compounds due to their high content of proteins, peptides, lipids, vitamins, minerals and other bioactive molecules [83]. These biological activities are likely associated with microbiome-mediated metabolite production, peptide release pathways and lipid-derived signaling molecules. Several insect-derived compounds exhibit antioxidant, antimicrobial, anti-inflammatory, and immunomodulatory properties, supporting their potential application in functional foods and health-related products [84].

In *H. illucens* larvae, antimicrobial peptide genes have been identified and functionally characterized, demonstrating potential relevance for pharmaceutical and antimicrobial applications [76]. In addition, *H. illucens* is considered a sustainable source of proteins, lipids, and bioactive compounds with industrial and biomedical potential [35]. The growing scientific interest in edible insects has also stimulated research into processing technologies and extraction methods aimed at improving the recovery and functionality of insect-derived bioactive compounds [31].

However, despite the documented bioactive potential, several limitations remain regarding safety, allergenicity, bioavailability and regulatory approval. Most current evidence is based on compositional screening and in vitro assays rather than mechanistic studies, bioavailability assessments, or clinical validation. Future multidisciplinary research should integrate mechanistic, preclinical, and clinical studies to clarify the therapeutic potential, long-term safety, and functional efficacy of insect-derived nutraceutical and pharmaceutical compounds.

### 6.4. Functional Ingredients and High-Value Biomolecules

Edible insects are increasingly explored as sources of functional ingredients and high-value biomolecules for food and biotechnological applications. Insect-derived proteins possess important techno-functional properties, including emulsifying capacity, foaming ability, and water-holding properties, supporting their incorporation into functional food formulations [32].

Bioactive peptides generated through protein digestion have demonstrated antioxidant and anti-inflammatory activities, suggesting potential nutraceutical applications of insect-derived compounds [83]. Furthermore, enzymatic hydrolysis has been shown to improve the techno-functional properties of insect proteins, enhancing their applicability in food processing systems [38]. Table 3 highlights that most microbiome studies focus on *H. illucens*, whereas evidence for other edible insect species remains comparatively limited. The table also illustrates the predominance of sequencing-based approaches over functional validation studies, emphasizing an important research gap.

However, despite promising functional properties, current research remains limited regarding large-scale extraction, purification efficiency, and the stability of insect-derived biomolecules. Moreover, variability among insect species and processing methods continues to affect product consistency and industrial applicability. Future studies should integrate technological optimization, mechanistic investigations, and scalable processing strategies to improve the production and functional utilization of high-value biomolecules from edible insects. Engineering strategies linking nutritional, microbiome, metabolic, and process-level interventions with targeted industrial applications are summarized in Table 4.

### 6.5. Safety, Stability, and Quality Control

Safety and quality control remain critical challenges for the industrial development of edible insect-based products. Microbial dynamics during insect production can significantly influence product safety, shelf stability, and processing quality. The industrial-scale production of lesser mealworms has demonstrated substantial changes in microbial communities throughout the rearing and processing stages, highlighting the importance of microbiological monitoring systems [37]. Similar microbial shifts have also been observed during *H. illucens* larval production, potentially affecting production efficiency and product stability [17].

Processing and formulation strategies also influence the microbiological quality of insect-derived foods. Bread enriched with *A. domesticus* powder showed acceptable nutritional and technological properties, although microbial stability and processing conditions remained important considerations for product safety [85]. Moreover, high-throughput sequencing analyses revealed diverse microbial communities in processed edible insects commercialized for human consumption, emphasizing the need for standardized hygiene and quality control procedures [69].

Despite increasing regulatory attention, several uncertainties remain regarding allergenicity, pathogenic microorganisms, substrate contamination, and long-term food safety. Potential biological hazards associated with edible insects also include parasites and other food safety risks that may arise when insects are harvested from uncontrolled environments or produced under inadequate hygienic conditions. These risks have been comprehensively reviewed by Grabowski et al. [86]. The European Food Safety Authority (EFSA) highlighted the necessity of risk assessment and standardized production practices for edible insect systems [87]. Although edible insects are generally considered safe when produced under controlled conditions, isolated cases of species-specific toxicological effects have been reported. For example, consumption of the African silkworm *Anaphe venata* has been associated with seasonal ataxia under particular nutritional conditions (Adamolekun et al. [88]), whereas the edible bug *Aspongopus nepalensis* contains unusual biochemical constituents that warrant species-specific safety assessments (Chakravorty et al. [89]). These reports highlight the importance of appropriate species selection, substrate control, and food safety monitoring. Therefore, further research is required to improve microbial safety, stability, traceability, and regulatory harmonization in edible insect production chains.

## 7. Current Challenges and Critical Limitations

### 7.1. Lack of Standardization

The lack of standardization remains one of the major limitations affecting the development of edible insect production systems. Considerable variability exists among studies regarding insect species, feeding substrates, rearing conditions, processing technologies, and analytical methodologies, making comparisons and reproducibility difficult [4]. This variability directly affects nutritional composition, microbial stability, and the functional properties of insect-derived products.

Processing technologies and production systems also differ substantially across industrial and experimental studies. Traditional and innovative processing methods can significantly influence nutrient quality, bioavailability and product safety, yet standardized protocols are still limited [28]. Moreover, current production technologies for insect proteins remain heterogeneous, affecting scalability and the consistency of functional properties [90].

Although edible insects are increasingly recognized as promising alternatives for food and feed systems, the absence of harmonized regulatory frameworks and quality control procedures continues to limit industrial implementation [53]. Therefore, future research should prioritize standardized rearing, processing, and analytical protocols to improve reproducibility, safety, and the large-scale commercialization of edible insect products.

### 7.2. Species-Specific Variability

Species-specific variability represents a major challenge in edible insect research and industrial application. Nutrient allocation, growth performance, lipid profiles, and bioactive compound accumulation can differ substantially among insect species, limiting the generalization of experimental findings [91]. Such variability complicates the development of standardized nutritional and processing strategies for large-scale production systems.

Comparative analyses have shown significant differences between edible insects and conventional animal proteins regarding protein quality, FA composition, and micronutrient content [27]. Importantly, lipid profiles vary considerably among insect taxa, even under similar rearing conditions, reflecting strong species-dependent metabolic characteristics [39]. For instance, *H. illucens* larvae, for example, exhibit distinct nutritional and metabolic properties that support their suitability for animal feed applications [39].

However, most current studies focus on a limited number of commercially relevant species, particularly *H. illucens* and *T. molitor*. Consequently, knowledge regarding less-studied edible insects remains fragmented. Further comparative and mechanistic investigations are therefore required to better understand species-specific metabolic responses and improve the predictability and standardization of edible insect production systems.

### 7.3. Limited Mechanistic Understanding

Despite increasing research on edible insect systems, mechanistic understanding of host–diet–microbiome interactions remains limited. Most studies have demonstrated that gut microbiota composition is influenced by diet, gut region, and developmental stage in *H. illucens* larvae [15,16]. However, the causal relationships linking microbial dynamics with nutrient metabolism, growth performance, and bioconversion efficiency are still poorly characterized.

Recent investigations have highlighted the potential importance of insect-associated microorganisms in agricultural sustainability and metabolic regulation [92]. Furthermore, microorganisms associated with mass-reared edible insects may contribute to nutrient transformation, pathogen control, and substrate degradation, although their functional roles remain insufficiently understood [93]. Genomic studies of *H. illucens* larvae have also provided valuable insights into metabolic adaptation and waste bioconversion pathways [64].

Nevertheless, most available evidence remains descriptive and fragmented, with limited integration of microbiome, metabolomic, and host physiological data. Therefore, further mechanistic and multi-omics investigations are required to better understand microbial functions and support precision nutritional engineering in edible insect production systems.

### 7.4. Reproducibility and Scalability Constraints

Despite the growing interest in edible insect production systems, reproducibility and scalability remain significant challenges for industrial implementation. Variability in substrates, rearing conditions, environmental parameters, and processing technologies often leads to inconsistent nutrient assimilation and bioconversion efficiency, limiting reproducibility across studies and production systems [5]. Life cycle assessments have demonstrated the sustainability potential of *H. illucens* biomass production; however, large-scale implementation still faces operational and economic limitations [8]. Likewise, edible insect farming systems differ substantially across regions and production scales, particularly in emerging markets where infrastructure, technical expertise, and regulatory support remain limited [68].

Current evidence also suggests that the sustainable integration of insects into food and feed systems requires more standardized and scalable production models [7]. Nevertheless, challenges related to consumer acceptance, market development, and industrial consistency continue to affect commercialization potential [9]. Therefore, future research should prioritize harmonized production protocols, scalable bioprocess technologies, and integrated sustainability assessments to improve reproducibility and industrial feasibility in edible insect systems.

### 7.5. Regulatory and Ethical Challenges

Regulatory and ethical challenges remain major barriers to the large-scale commercialization of edible insects for food and feed applications. Current regulations differ considerably among countries, while standardized guidelines regarding production, processing, labeling, and safety assessment are still limited. The European Food Safety Authority (EFSA) has highlighted important concerns related to allergenicity, microbial contamination, substrate safety, and chemical hazards in edible insect systems [87].

Experimental data have demonstrated substantial microbial variability in commercially available edible insects and processed insect-based products [54,69]. In addition, microbial dynamics during industrial-scale production may affect product safety and shelf stability, emphasizing the importance of strict hygiene and quality control procedures [37]. Processing technologies can also influence microbiological safety, nutritional quality, and consumer acceptance of insect-derived foods [28].Consumer perception and ethical acceptance continue to represent additional limitations for market expansion. Cultural attitudes, food neophobia, and concerns regarding safety and processing remain important obstacles to the wider adoption of insect-based products [31]. Moreover, although edible insects contain promising bioactive compounds with potential health benefits, their biological activity, long-term safety, and regulatory classification remain insufficiently understood [94].

Future efforts should prioritize harmonized regulatory frameworks, standardized safety protocols, transparent labeling, and consumer education to facilitate the safe, ethical, and sustainable integration of edible insects into food and feed systems.

## 8. Future Perspectives and Emerging Engineering Approaches

Current limitations and future engineering priorities in edible insect biomass systems are summarized in Table 5.

### 8.1. Predictive Nutritional Engineering

Predictive nutritional engineering is emerging as a promising strategy for optimizing edible insect production through the integration of microbiome dynamics, dietary modulation, and processing technologies. Recent evidence suggests that insect-associated microbiota play central roles in regulating physiology, metabolism, and nutrient utilization, supporting the development of microbiome-guided production approaches [95]. In *H. illucens*, gut microbial communities respond dynamically to diet, gut region, and developmental stage, highlighting the complexity of host–microbiome interactions [15].

Insect-derived bioactive compounds are also attracting increasing attention for their potential functional and health-promoting applications. Chitin-derived prebiotics and bioactive peptides from *H. illucens* larvae may contribute to gut health modulation and nutraceutical development [96]. In parallel, advances in insect processing technologies are improving protein recovery, sustainability, and production efficiency, supporting the transition toward more optimized and scalable insect-based systems [97].

Nevertheless, predictive nutritional engineering remains limited by insufficient mechanistic understanding and the lack of integrated computational models linking microbiome composition, metabolic pathways, and production outcomes. Therefore, future research should combine omics technologies, precision feeding, and predictive modeling approaches to improve nutritional optimization and sustainability in edible insect systems.

### 8.2. Artificial Intelligence-Assisted Precision Feeding

Artificial intelligence (AI)-assisted precision feeding is emerging as a promising approach for optimizing edible insect production systems through data-driven nutritional and metabolic management. Advances in genomics and the genetic analysis of *H. illucens* have provided valuable datasets for understanding nutrient utilization, metabolic adaptation, and waste bioconversion pathways, creating opportunities for predictive feeding models and automated production optimization [64].

Recent metatranscriptomic studies have revealed key lignocellulolytic enzymes and functional microbial pathways involved in substrate degradation within *H. illucens* larvae gut microbiomes [98]. Such omics-derived information may support AI-based strategies capable of predicting substrate performance, microbiome responses, and nutrient conversion efficiency under different rearing conditions.

The growing expansion of edible insect farming, particularly in emerging regions, also increases the need for scalable and automated production systems [68]. Importantly, sustainability assessments have demonstrated the importance of optimizing feed conversion and resource utilization to improve environmental performance in industrial insect farming [8]. Despite their potential, current AI applications in edible insect systems remain limited because of the lack of standardized datasets and the limited integration of microbiome, metabolic, and production parameters. Future research should integrate artificial intelligence, omics technologies, precision feeding, and systems biology to develop robust predictive models for industrial-scale edible insect production.

### 8.3. Synthetic Microbiomes and Microbiome Engineering

Synthetic microbiomes and microbiome engineering are emerging as innovative approaches for improving nutrient conversion, metabolic efficiency, and health stability in edible insect systems. Recent studies suggest that insect gut microbiota are dynamically regulated by environmental conditions, host physiology, and dietary substrates, highlighting the ecological complexity of insect–microbe interactions [99]. In *H. illucens*, gut microbial communities vary according to gut region, developmental stage, and diet composition [15].

Core microbial taxa associated with *H. illucens* larvae have also been identified under controlled rearing conditions, suggesting the existence of relatively stable microbiome structures linked to nutrient metabolism and substrate degradation [58]. Furthermore, microbial community dynamics during larval rearing may directly influence exploitation potential, growth performance, and bioconversion efficiency [17]. These findings support the possibility of designing targeted microbial consortia for improving industrial insect production systems.

Despite growing interest in microbiome engineering, current knowledge regarding functional microbial interactions and host regulatory mechanisms remains limited. Recent reviews have emphasized the complexity of *H. illucens* microbiome interactions and the need for more mechanistic and integrative investigations [100]. Therefore, future research should focus on synthetic microbiome design, functional validation, and microbiome-guided precision feeding strategies to improve sustainability and predictability in edible insect production systems.

### 8.4. Metabolomics, Multi-Omics and Systems Biology

**Figure 4:**
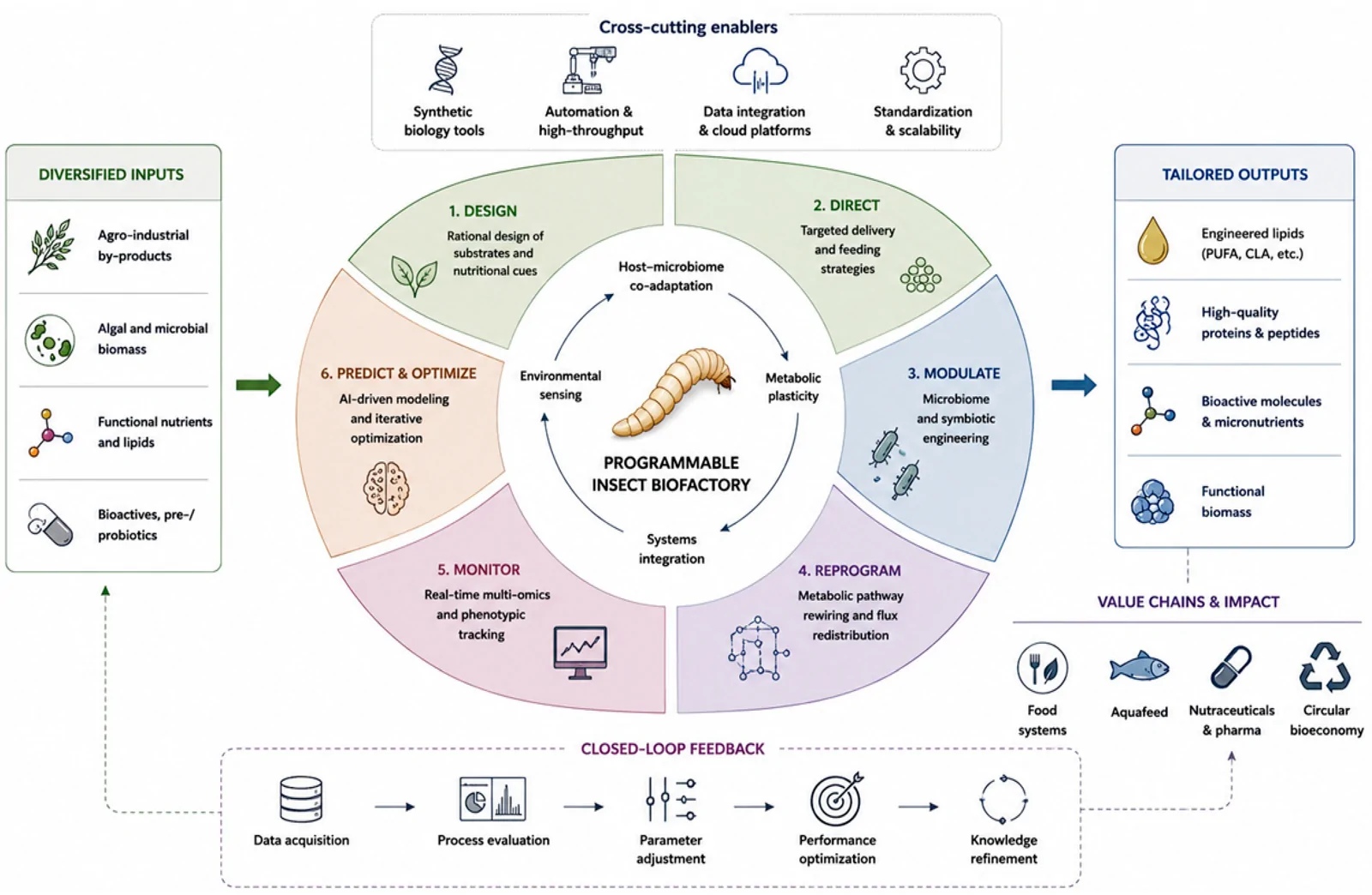
Integrated systems engineering roadmap for programmable insect biofactories.

An integrated systems-oriented engineering roadmap for programmable insect biofactories is proposed in Figure 4. Metabolomics and multi-omics approaches are increasingly advancing the understanding of complex host–diet–microbiome interactions in edible insect systems. Recent studies integrating gut microbiome analysis with transcriptional profiling have demonstrated that dietary substrates significantly influence microbial communities and gene expression patterns in *H. illucens* larvae [101]. These findings highlight the importance of systems level analyses for understanding metabolic adaptation and nutrient conversion mechanisms.

Genomic investigations of *H. illucens* have also provided valuable insights into genes associated with waste biodegradation, nutrient metabolism, and physiological regulation [64]. In addition, microbiome-associated metabolic functions were shown to enhance food waste biodegradation efficiency in *H. illucens* larvae, reinforcing the role of gut microorganisms in substrate conversion processes [61]. Functional bacteria combined with *H. illucens* larvae further improved biowaste conversion into protein-rich biomass and organic fertilizer products [62].

Despite these advances, integration of metabolomics, transcriptomics, microbiomics, and physiological data remains limited in edible insect research. Current studies are often fragmented and lack standardized analytical frameworks. Recent evaluations have emphasized that microorganism contributions to mass-reared edible insects remain insufficiently characterized at functional and systems-biology levels [93]. Therefore, future research should prioritize integrated multi-omics and systems biology approaches to improve predictive modeling, microbiome engineering, and precision nutritional optimization in edible insect production systems.

### 8.5. Programmable Insect Biofactories

Programmable insect biofactories are emerging as promising systems for converting organic waste into high-value proteins, lipids, and bioactive compounds. *H. illucens* is particularly relevant due to its high bioconversion efficiency and adaptability to diverse waste substrates [35,79]. *H. illucens* larvae can efficiently degrade complex organic materials, supporting sustainable circular bioeconomy applications [5].

Beyond biomass production, edible insects represent valuable sources of bioactive compounds with nutraceutical and industrial application [84]. However, current insect biofactory systems remain largely empirical and poorly standardized. Important limitations persist regarding metabolic regulation, microbiome control, scalability, and biosafety. Therefore, future progress will require integrated approaches combining precision nutrition, microbiome engineering, multi-omics, and artificial intelligence to develop more controllable and scalable insect bioproduction systems.

### 8.6. Smart Insect Farming and Automated Bioprocessing

Smart insect farming and automated bioprocessing are increasingly viewed as essential for improving the efficiency, sustainability, and scalability of edible insect production systems. Recent technological advances in insect rearing and processing have focused on automation, environmental monitoring, and optimized biomass conversion to support industrial-scale production [102]. Edible insect farming is rapidly expanding as a sustainable enterprise, particularly in emerging regions where insects may contribute to food security, waste valorization, and circular bioeconomy strategies [68]. In addition, life cycle assessments have demonstrated that *H. illucens* biomass production can reduce environmental impacts compared with conventional protein systems [8].

Yet, important limitations remain regarding process standardization, automation costs, operational reproducibility, and biosafety control. Current systems are still highly dependent on variable substrates and environmental conditions, limiting industrial consistency. Future smart farming platforms should integrate automation, artificial intelligence, sensor technologies, digital monitoring, and precision bioprocessing to improve productivity, process reproducibility, and sustainability in edible insect production systems.

## 9. Conclusions

Edible insects are increasingly recognized as advanced biological platforms with significant potential for sustainable food production, waste valorization, and circular bioeconomy systems. Current research demonstrates that diet composition, gut microbiota, and metabolic regulation strongly influence nutrient conversion, lipid metabolism, growth performance, and bioactive compound accumulation.

Nevertheless, despite substantial progress, major limitations still restrict the transition from experimental insect systems to scalable industrial bioprocesses. Current studies remain largely descriptive, while the mechanistic understanding of host–diet–microbiome interactions, metabolic pathways, and microbial functionality is still incomplete. Importantly, the absence of standardized rearing protocols, predictive models, automated processing systems, and harmonized regulatory frameworks continues to limit reproducibility, biosafety, and industrial scalability.

Future development of edible insect biotechnology will therefore require a stronger integration of metabolic engineering, microbiome engineering, precision feeding, systems biology, and smart bioprocess engineering. The integration of artificial intelligence, multi-omics, and automated bioprocessing will facilitate the development of programmable insect biofactories. Consequently, edible insects should be considered not only as additional protein sources, but also as engineerable biological systems with substantial potential for next-generation sustainable biomanufacturing and industrial biotechnology.

## Figures and Tables

**Table 1:** Nutritional composition across selected edible insect species.

Species	Protein (%)	Lipid (%)	Major FAs	Functional/Bioactive Components	Main Applications	References
*T. molitor*	47–60	28–38	Oleic acid (18:1n−9), linoleic acid (18:2n−6), palmitic acid (16:0)	Bioactive peptides, antioxidants, chitin	Functional foods, aquafeed, protein ingredients	[11,27]
*A. domesticus*	55–70	15–25	Oleic acid, linoleic acid, α-linolenic acid (18:3n−3)	Antioxidant peptides, micronutrients, minerals	Human nutrition, protein-enriched foods	[29,31,33]
*H. illucens*	35–55	20–40	Lauric acid (12:0), oleic acid, palmitic acid	Antimicrobial peptides, functional lipids, chitin	Animal feed, waste bioconversion, industrial biomass	[34,35,36]
*Alphitobius diaperinus*	50–65	20–30	Oleic acid, linoleic acid, palmitic acid	Protein concentrates, bioactive compounds	Feed applications, functional ingredients	[27,,37]
*Locusta migratoria*	50–65	10–20	Oleic acid, linoleic acid	Functional proteins, minerals	Functional foods, alternative proteins	[38,39]
*Gryllus bimaculatus*	58–71	12–22	Oleic acid, linoleic acid, α-linolenic acid	Bioactive peptides, minerals	Human food, nutraceutical ingredients	[31]

**Table 3:** Gut microbiome composition and metabolic functions in edible insects.

Species	Dominant Microbial Taxa	Main Metabolic Functions	Diet-Related Microbiome Modulation	Physiological/ Metabolic Impact	References
*H. illucens*	*Enterococcus*, *Providencia*, *Morganella*, *Klebsiella*	Organic matter degradation, lipid metabolism, nutrient assimilation	Strongly influenced by substrate composition and waste-derived diets	Regulation of lipid accumulation and bioconversion efficiency	[16]
*T. molitor*	*Spiroplasma*, *Lactococcus*, *Enterobacteriaceae*	Carbohydrate metabolism, digestion, metabolite production	Modulated by cereal-based and algal diets	Altered nutrient partitioning and FA metabolism	[69]
*A. domesticus*	*Lactobacillus*, *Pantoea*, *Bacillus*	Protein digestion, immune regulation, metabolite synthesis	Influenced by plant-derived substrates and dietary supplements	Improved digestive efficiency and metabolic performance	[70]
*A. diaperinus*	*Enterococcus*, *Bacillus*, *Staphylococcus*	Nutrient degradation and microbial fermentation	Sensitive to rearing substrate composition	Effects on microbial stability and nutrient conversion	[37]
*H. illucens*	Core microbiota associated with waste conversion systems	Detoxification and biotransformation processes	Waste-stream composition reshapes microbial communities	Enhanced waste valorization and biomass productivity	[58]
*H. illucens*	Functional microbial consortia	Metabolic signaling and nutrient routing	Microbiome shifts induced by dietary lipid supplementation	Regulation of metabolic fluxes and FA biosynthesis	[22]
*T. molitor*	Gut-associated fermentative bacteria	Short-chain metabolite production and digestive assistance	Influenced by agro-industrial by-products	Functional modulation of nutrient utilization	[71]

**Table 4:** Engineering strategies and industrial applications of edible insect biomass.

Engineering Strategy	Technological Approach	Targeted Metabolic/ Nutritional Outcome	Industrial Application	Major Limitations	References
Precision feeding	Tailored substrate formulation and nutrient balancing	Controlled lipid accumulation and optimized protein composition	Functional foods and aquafeed production	Limited standardization among species and diets	[14,78]
Agro-industrial waste valorization	Conversion of organic residues into insect biomass	Sustainable nutrient recycling and waste bioconversion	Circular bioeconomy and waste management	Variability in substrate composition	[5,79]
Algal biomass supplementation	Integration of microalgae and macroalgae into diets	Omega-3 enrichment and PUFA modulation	Nutraceutical and functional lipid production	High production costs and scalability issues	[18,41]
Microbiome engineering	Modulation of gut microbial communities and functional microbiota	Enhanced nutrient assimilation and metabolic regulation	Engineered biomass optimization	Limited mechanistic understanding	[22,58]
Multi-omics integration	Metagenomics, metabolomics and transcriptomics approaches	Systems-level understanding of host–microbiome interactions	Predictive nutritional engineering	High analytical complexity	[64,71]
AI-assisted optimization	Machine learning and predictive modeling	Data-driven regulation of nutritional outputs	Smart insect farming systems	Lack of integrated datasets	[78]
Metabolic engineering	Manipulation of lipid biosynthesis and nutrient partitioning pathways	Tailored production of bioactive compounds and functional lipids	Pharmaceutical and nutraceutical applications	Limited pathway characterization	[64]
Automated bioprocessing	Digital monitoring and industrial-scale insect production systems	Enhanced productivity and process scalability	Industrial biomass production	Technological and economic constraints	[68]

**Table 5:** Current knowledge gaps and future engineering priorities in edible insect biomass systems.

Current Limitation	Scientific/Technical Challenge	Engineering Priority	Future Research Direction	References
Lack of standardized feeding protocols	High variability among diets, substrates, and rearing systems	Development of precision feeding frameworks	Harmonized nutritional engineering strategies across insect species	[4,14]
Limited mechanistic understanding of host–microbiome interactions	Poorly resolved metabolic pathways and microbiome functionality	Systems-based metabolic regulation models	Integration of microbiome engineering and metabolic flux analysis	[22,71]
Species-specific nutritional variability	Biological heterogeneity among edible insect species	Predictive nutritional engineering	Comparative systems biology approaches	[27]
Limited scalability of industrial insect farming	Process instability and technological limitations	Automated bioprocess engineering	Smart insect farming systems and digital monitoring	[68,78]
Insufficient integration of omics technologies	Fragmented datasets and analytical complexity	Multi-omics integration platforms	AI-assisted predictive modeling and systems biology	[64,71]
Incomplete characterization of lipid biosynthesis pathways	Limited understanding of FA regulation and nutrient partitioning	Metabolic engineering of functional lipids	Tailored omega-3 and PUFA enrichment strategies	[18]
Regulatory and safety uncertainties	Lack of harmonized legislation and quality standards	Development of regulatory frameworks and quality control systems	International standardization for edible insect biomass	[87]
Limited transition from descriptive to predictive studies	Most studies remain empirical and species-specific	Data-driven nutritional engineering	Development of programmable insect biofactories	[5,64]

## Data Availability

No new data were created or analyzed in this study. Data sharing is not applicable to this article.
